# Authentication Protocol for Cloud Databases Using Blockchain Mechanism

**DOI:** 10.3390/s19204444

**Published:** 2019-10-14

**Authors:** Gaurav Deep, Rajni Mohana, Anand Nayyar, P. Sanjeevikumar, Eklas Hossain

**Affiliations:** 1 Department of CSE & IT, Jaypee University of Information Technology, Solan 173234, India; deepgaurav48@gmail.com (G.D.); rajni.mohana@juit.ac.in (R.M.); 2 Graduate School, Duy Tan University, Da Nang 550000, Vietnam; 3 Department of Energy Technology, Aalborg University, 6700 Esbjerg, Denmark; 4 Oregon Renewable Energy Center (OREC), Department of Electrical Engineering and Renewable Energy, Oregon Tech, Klamath Falls, OR 97601, USA; eklas.hossain@oit.edu

**Keywords:** cloud computing, cloud databases, insider threat, outsider threat, access control, Blockchain, cluster, hash value, claims

## Abstract

Cloud computing has made the software development process fast and flexible but on the other hand it has contributed to increasing security attacks. Employees who manage the data in cloud companies may face insider attack, affecting their reputation. They have the advantage of accessing the user data by interacting with the authentication mechanism. The primary aim of this research paper is to provide a novel secure authentication mechanism by using Blockchain technology for cloud databases. Blockchain makes it difficult to change user login credentials details in the user authentication process by an insider. The insider is not able to access the user authentication data due to the distributed ledger-based authentication scheme. Activity of insider can be traced and cannot be changed. Both insider and outsider user’s are authenticated using individual IDs and signatures. Furthermore, the user access control on the cloud database is also authenticated. The algorithm and theorem of the proposed mechanism have been given to demonstrate the applicability and correctness.The proposed mechanism is tested on the Scyther formal system tool against denial of service, impersonation, offline guessing, and no replay attacks. Scyther results show that the proposed methodology is secure cum robust.

## 1. Introduction

Data security has turned into significant concern because of the massive development of cloud computing and networks. Therefore, methods that shield the information from fabrication, interception, and modification have turned out to be a critical issue. A large amount of data is stored in the cloud database. The users can store, modify and retrieve the data anywhere in the world. Therefore, it is essential to secure privacy in a cloud databases [1]. According to the Information security breaches survey (ISBS), 2015 large organizations stated that there was an element 81% of staff involved in some of the breaches they suffered [2], 90% of organizations feel vulnerable to an insider threat according to the Insider Threat 2018 Report [3] and Forrester Research [4].

Insider threat is the most perilous threat that harms various organizations like Yahoo, Facebook, and Google. Richardson et al. [3] proved that the expense of the data records lost in insiders attack is more prominent than the expense of those lost to outsiders. This is because insiders know about the system framework and attack the profitable records, while outsiders take that information which is accessible [5,6]. According to the 2016 U.S. State of Cybercrime Survey [7], insiders are answerable for 27% of all electronic crimes. This survey also revealed that nearly one-third of the respondents thought that damage caused by insider attacks was more severe than the damage caused by outsider attacks.

The number of insiders may increase due to the transfer of data over the cloud, which leads to more insider threats. Additionally, new security systems are required to secure unauthorized data from the insiders because the insider knows how and where data ensured in the organization. Previously various algorithms have been used to secure the data from insider threat on the cloud. However, those algorithms do not secure the data from certified users who misuse their rights to violate the security of the system. Therefore, designing such an algorithm that can secure the data from insiders has turned into a critical demand because of the damage that can be induced by the insiders.

In literature, researchers have worked on other security issues like outside malicious attacks, access control issues, network breaches, data provenance, resource exhaustion, consistency management, etc. However, much less work has been proposed on anticipating insider attacks [1,8,9,10,11,12,13,14,15,16,17,18,19,20,21,22,23], which is the primary objective of this study.

The existing user authentication techniques fail to secure the data from the insiders, due to the following loopholes: (1) The password of the user can be guessed easily by the insider. (2) The two-factor authentication used by Google authenticator (GA) to send codes to the user via Short Message Service is also not secure as the code sent on Short Message Service can be cracked by the attacker due to a security breach that could lose all user authentication codes [24]. (3) In the case of GA and other third-party authentication applications (TPAA), all the authentication codes are owned by a single identity that makes it more vulnerable [25].

### 1.1. Motivation

The research paper uses Blockchain mechanism as it is open to the public to resolve the above mentioned loopholes. Blockchain uses a decentralized approach, in which the chain is fully open to the public, and no sensitive data is stored. It is not possible for an insider to make changes in the user’s authentication data. To do changes in any existing node of Blockchain, all its previous nodes need to be changed. The services of cloud database which are accessible by the end-user is also authenticated with Blockchain mechanism.

### 1.2. Research Contribution 

A novel authentication algorithm proposed for managing the insiders on the cloud by blockchain based authentication mechanism. The proposed work makes the following contribution:

- The proposed mechanism is authenticating the insider as well as outsider attack on the system.
- The peer-to-peer authentication is provided to the cloud database user via Blockchain mechanism.
- The performance of the system is evaluated via formal system tool—Scyther and results demonstrate that the proposed mechanism is robust and secure.

The research paper is organized as follows- Section 2 presents the literature review of various prevention techniques against insider and outsider threats. Section 3 highlights the proposed authentication mechanism for insiders and cloud users. Section 4 includes the verification of the proposed methodology by using verification tool-Scyther and finally, the paper is concluded in Section 5.

## 2. Related Works

Previous researchers have proposed various techniques on insider and outsider threats over the cloud, but still there is a need to work on both threats over a cloud database. Therefore, related work is divided into insider and outsider threats. 

### 2.1. Insider Threat 

Previously researchers have worked on behavioral analysis to develop authorization policies for insiders. Some researchers have designed a Crypto Processor to integrate insider with a particular system. Therefore, this section presents the work done on insider threats in cloud databases. Table 1 presents a comparison of seven approaches that are proposed for Insider Threat. 

Wu et al. [1] observed the encryption technique to prevent understanding of user data. Before applying the query on the user data, it should be decrypted first and after finishing the query process, the data is again encrypted. Therefore, to prevent the tedious task of encryption–decryption–encryption, author proposed a feature index technique which extracted the features from the user data before encryption and the querying process done on the cloud. The encryption was undertaken with an index generator; a feature index of user data which was prepared with the help of query translator and query executor, further the technique executed the query with the help of a feature index. 

Moon et al. [8] introduced the insider behavior analysis server. In this study, they proposed two-tier architecture using cloud and In-Memory Database (IMDB) for a database protection system. The work done by the insider is stored in audit logs which was further sent to file and database log pre-processor. After that, the log data was pre-processed and the data is sent to the insider behavior analysis (IBA) server. The IBA server detected the presence of attack and incorporated the cloud capability.

Yaseen et al. [9] discussed the prevention measures of insider threat prediction. The author proposed the knowledgebase algorithm with the advantage of Constraint and Dependency Graph, Neural Dependency and Inference Graph, hot cluster, safe cluster, and dependency matrix. The knowledge graph is generated for predicting the insider attack using the proposed algorithm. Yaseen et al. [10] designed the threat prediction graph using the knowledgebase algorithm in extended work. Threat prediction value of each data, available in knowledge graph of insider is represented by threat prediction graph(TPG) and helped in predicting and preventing insider attack.

Yaseen et al. [11] proposed another work on insider threat. In this study, the author proposed Policy Enforcement Point (PEP)-Policy Decision Point (PDP) architecture by using the Knowledgebase algorithm and database dependency checker. The proposed system was tested with multiple Policy PEPs and a single PDP. The accuracy of the proposed system is enhanced when the PEPs number is less.

Dou et al. [12] drafted the trusted platform module-based authentication protocol for Hadoop for removing the Kerberos limitations in terms of user authentications and insider attacks. In this proposed work, authentication keys and authentication operations were locally hidden. The trusted platform module store the current software and hardware details of the hosting machine in an internal set of platform configuration registers. The proposed protocol could be bound for specific systems securing them against the insider attacks.

Shaghaghi et al. [13] designed the extended version of access control architecture called Gargoyle Software-Defined Network (GSDN) architecture based on Crampton and Huth’s architecture to detect and deter suspicious activities of an insider. Further, the author retrieved contextual information by passively analyzing network traffic. The GSDN has three main components: context analyzer, risk management, and advanced enforcement point. The proposed work covered network traffic monitoring to extract insider activity details. From this, the various risks gets detected, and actions were taken on various user authorizations.

Chattopadhyay et al. [14] implemented a time-series classification approach for insider activities which helped in detecting insider threat. The analysis of insider behavioral was done by tracking single-day features and over time. The features vectors of each single-day statistic and over a period constructed. These features judged a malicious or non-malicious insider. Classification (a two-layered deep auto-encoder neural network) is done to improvise the results. 

Baracaldo et al. [15] developed Geo-Social Insider Threat-Resilient Access Control Framework (G-SIR) monitors to detect the insider activities by the movements. Furthermore, it classified attackers into enablers, inhibitors or neutral. Inhibitors defined as risky users, enablers increased the trust and neutral users neither increased nor decreased the risk. It used Policy Enforcement Point (PEP)-Policy Decision Point (PDP) model along with monitoring, context, and inference and access control module. The permissions and roles were written in role-based access control (RBAC).

### 2.2. Outsider Threat 

Many researchers have worked on authenticating an outsider on the cloud. Table 2 presents comparison of many approaches based on outsider threat. The user authentication from accessing the cloud services plays a significant role in restricting the various hackers and attacks so that legitimate user’s can access the data.

Tsai et al. [16] proposed a user authentication scheme for distributed mobile cloud-computing services based on Elliptic curve cryptography. The proposed sceheme is used to authenticate mobile users to access cloud computing services from multiple service providers who use only a single private key. It included three entities: user, smart card generator and service provider. First, the user and service provider gets registered with smart card generator where public and private keys generated for them. Therefore, they can authenticate each of them without the involvement of the Smart card generator. The scheme provides mutual authentication, key exchange, user anonymity, and user intractability.

Yang et al. [17] designed the two-factor authentication protocol with open ID for accessing data on the multimedia cloud using the Diffie–Hellman algorithm. In this protocol, smart card along with user login details allowed the multimedia data accessing to the whole family. The multiple cloud models were used for various purposes like smart card authentication, user credentials authentication, multimedia data cloud, etc. The authorization policies are written in Role-based access control (RBAC) to validate the protocol proposed. Further, three analysis namely secure analysis, functional analysis and efficiency analysis (time complex and message exchange time to compare with other research) were also undertaken.

Kumari et al. [18] developed a multi-factor authentication for IoT and cloud servers with the use of login, cookies and device details. The proposed work handled the limitations of existing work like offline password guessing, insider attack, absence of device anonymity and no session key computation by using a temper-resistant device, elliptic curve cryptography, etc. This authentication protocol was found suitable for resource constraint Internet of Things (IoT) where mutual authentication was required.

Shajina and Varalakshmi [19] proposed a multi-owner authentication protocol that took the multiple owners in a cloud for authentication by using Triple Data Encryption Standard. This protocol increased the security requirement of single sign on by using the dual authentication of a group manager and service manager. A primary owner in a group can add other owners in a group along with their access permissions. Furthermore, certification authority verified the credentials of owners and provide them a valid token with name, expiration time, services required, etc. These services were accessed by getting session tokens from a session manager and precedence-based access control lists stored in a cloud server.

Anakath et al. [20] observed that a trust model for authentication played an important role where device identity was identified and an authentication protocol was selected. The three factors used for an authentication purpose were knowledge, possession, and inherence. This protocol used the possession factors, one-time password and passwords which were known by users only. The user details were stored in big data which uses Privacy-Preserving Multi-factor Cloud Authentication System where the user profile was created that stored various user parameters in encrypted form by using simple-homomorphic encryption.

Chaudhry et al. [21] improved the user authentication scheme for distributed mobile cloud computing services by developing the authenticating schema for mobile users by using elliptic curve cryptography (ECC). The proposed scheme allow users to access cloud computing services from multiple service providers by using a single private key. The methodology improved the authentication phase to prevent server forgery attack and was validated in ProVerif automatic cryptographic protocol verifier, which showed that the proposed work being more secure and robust as compared to the work of Tsai et al. [16].

Kumar et al. [22] proposed a biometrics-based recognition (face features) system for authenticating cloud users by using elliptic curve cryptography. The system extract the facial features of cloud users being stored in a cloud biometric database in encrypted form. Initially, it acquire the face images; after that images are pre-processed and facial features are extracted, and in the last step, the recognition is performed using an encrypted biometric feature. The recognition step of cloud users was done by matching the similarity scores of facial features.

Neha and Chatterjee [23] designed a biometrics-based re-authentication system that utilized the fixed text keystroke dynamics. The system enhanced the security level over the traditional password-based authentication mechanism. The authentication process consisted of keystroke dynamics enrolment, identification and verification factors. In this user name and password was asked from the user and system captured typed rhythm. These features were stored in the database and later extracted by a k-means clustering algorithm. The experiment was conducted on three types of data sets (heterogeneous, homogeneous, and aggregate feature sets).

From Table 1 and Table 2, it can be seen that many protocols are designed and implemented to combat insider, outsider attacks as well as attacks inside the cloud. Staff members of cloud service providers manage the user data on the cloud. These staff members enjoy the highest privileges for data management. These staff members work as insiders to cloud service providers. Insider activities can monitor by first applying authentication; once an insider is authenticated, it is easy to monitor and track his activities. There is a need to apply authentication policy which is not changeable and accessible by insiders themselves.

Furthermore, the user data stored over the cloud should not be accessible by an attacker, and it is accessible to only a genuine user. The previous researchers introduced the user authentication control authority or multi-factor authentication policies to a complex system. There is a need to introduce a distributed ledger-based authentication policy which works on the peer-to-peer basis.

It is evident from Table 1 and Table 2 that no work was done on insider authentication by using Blockchain mechanism. Attacker takes advantage of controlling user and insiders data in existing techniques.The responsibility can be fixed if any insider threat is posed, and the insider should not be allowed to change its authentication details to save himself from tracking after commiting insider attack. For any cloud user authentication is must, user may be any device or human being. If a third party providing authentication is breached, its purpose is nullified. To make things difficult for an attacker, a distributed ledger-based authentication scheme is proposed for the outside user, where it is not easy to change every ledger entry that is stored in a distributed manner by using Blockchain. So, there is a requirement of authentication protocol which cannot be altered by anyone, including an insider and outsider.

## 3. Proposed Blockchain Authentication Mechanism (BAM)

This section explains the proposed authentication policies and Blockchain authentication protocol for an insider as well as a database cloud user.

### 3.1. Blockchain Mechanism

**Figure 1:**
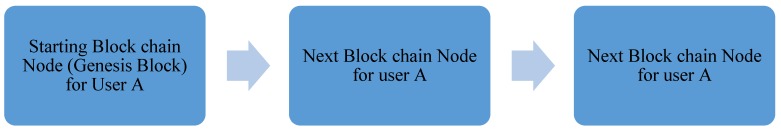
Blockchain starting from new node (genesis block).

Zheng et al. [25], discussed the importance of the Blockchain mechanism. The author suggested that Blockchain helps in removing the limitations of many applications in existing technologies and increased system performance. Furthermore, the author observed that Blockchain was also useful in user authentication applications. The Blockchain uses Blockchain ID, which is bounded with a public key, and transferred the ownership of the private key to the intended user. The user signatures helped in verifying against the public key which is stored in the Blockchain ID. Minoli et al. [26] utilized the Blockchain at various security levels in an IoT-based health care system. The author noticed that Blockchain was resistant in modifications to existing data in a linked list of blocks. It removed the concept of a trusted third party for the authentication process. Furthermore, it worked as peer-to-peer in distributed systems, where the peer-supported state of a distributed ledger and network has no central control. The Blockchain mechanism is based on a decentralized approach, which provides numerous benefits over traditional authentication methodologies. It helps in tracking the previous records and activities of the user. For example, the current user-authenticated node is connected to the previous node as so on up to the starting node [27,28,29] as shown in Figure 1.

Each Blockchain node further consists of elements working on many parameters. The first node/starting node of the Blockchain is known as the genesis block, and the node value of the index and previous hash are set as zero. The Timestamp records the time of node creation, and Predefined value stored in Current Hash value. The index value notified the position of the current block node in the chain.

The length of the hash value fixed and its alphanumeric value uniquely identifies the data or the digital data fingerprints. The first three digits of a valid hash should be zero. Furthermore, the same data value always mapped to the same hash value. It is computationally infeasible to convert hash value to data value. The current hash value is calculated by using a hashing function, as described in Equation (1).

(1) Hashing fuction (Index+Previous Hash value+Timestamp+Data+Nonce value)=Current Hash value

The nonce value is used to find a valid hash. Therefore, it is required to find a nonce value that produced a valid hash when used with the rest of the information from that block.

Next, the user’s credentials’ information is stored in the cloud to authenticate users on the database cloud. The Blockchain is used to prevent any user data leakage. The user’s login detail saved in a cloud database which is authenticated in peer-to-peer architecture on the cloud database at various levels. Blockchain finds many applications in various areas [30,31,32,33,34,35,36,37,38,39,40,41,42,43,44,45,46].

### 3.2. Overall Framework

#### Algorithm and Theorem

Algorithm 1 highlights the essential steps of the proposed Blockchain authentication mechanism for cloud database. It covers both insider and outsider user. As demonstrated in the algorithm, initially it checks for user credentials, then checks for valid Blockchain node parameters. If all goes well, then the user gets authenticated. If the user’s credentials information does not exist in the cloud database, then the user is asked for retrying or for new user account creation.

**Algorithm 1** User Authentication using Blockchain Mechanism.Input: Request Q received at Blockchain Database Server/Cloudb, It checks for Q Request is from an insider (Bob) or an outsider. Output: Access Granted or Rejected.Step 1: If Request == Insider (Bob) Go to Step 2 else Go to step 5Step 2: If Login ID &User Signature== Valid then continue this step else Go to Step 3If current index value > Last stored index ˄Hash value ˄ Timestamp value˄ Nonce value == Valid then continue this step else Go to step 4.Create New Blockchain node and Grant Authentication.Step 3: If User ≠ ≠ Exist in Blockchain Database then for Retrying Go to Step 1 else continue this step    Add new user Node (Genesis Block)       Initialize Index value       Allocate current Time stamp value      Store Predefined value in Current Hash value      Store Data value      Allocate valid Nonce Value  Update user record in Blockchain DatabaseStep 4:   Give error message and ExitStep 5:   If User== Outsider Go to Step 2 else go to Step 3

**Proof** **of** **Algorithm** **Correctness.** The following theorem proves that the user is authenticated using Blockchain.

**Theorem** **1.** *All authentication conditions of the Blockchain are met if and only if, a user authenticated.* □

**Proof.**  If all authentication conditions of the Blockchain met, the user authenticated.

P → Q(2)

here in this statement P is “all authentication conditions of the Blockchain are met” which implies Q “user is authenticated”.

If the user is authenticated then all authentication conditions of the Blockchain were met

Q → P(3)

here in this statement Q is “user is authenticated” which implies “all authentication conditions of the Blockchain were met”

It means P are Q are in bi-conditional statement P ↔ Q for this to be true either one of the statement should be true.

If all authentication conditions of the Blockchain are met, the user is authenticated.

(4) (P ∧Q)

or

If all authentication conditions of the Blockchain were not met then the user is not authenticated.

(5) (¬P∧¬Q)

(6) P↔Q≡(P∧Q)∨(¬P∧¬Q)

Here it can be seen that Left Hand Side is logically equivalent to Right Hand Side, it can be proved by taking Left Hand Side and deriving it.

(7) P↔Q≡(P→Q)∧(Q→P)

(8) ≡(¬P∨Q)∧(¬Q∨P) (NEGATING THE HYPOTHESIS)

(9) ≡[(¬P∨ Q)∧¬Q]∨[(¬P∨Q)∧P] (LAW OF DISTRIBUTIVE)

(10) ≡[(¬P∧¬Q)∨(Q∧¬Q)]∨[(¬P∨Q)∧P] (LAW OF DISTRIBUTIVE)

(11) ≡[(¬P∧¬Q)∨F]∨[(¬P∨Q)∧P]

(12) (INVERSE LAW P∧¬P≡FAND P∨F≡P IDENTITY LAW).

(13) ≡(¬P∧¬Q)∨[(¬P∨Q)∧P]

(14) ≡(¬P∧¬Q)∨[(¬P∧P)∨(Q∧P)] (LAW OF DISTRIBUTIVE)

(15) ≡(¬P∧¬Q)∨[F∨(Q∧P)]

(16) (INVERSE LAW P∧¬P≡F AND P∨F≡P (IDENTITY LAW)

(17) ≡(¬P∧¬Q)∨(Q∧P)

(18) ≡(¬P∧¬Q)∨(P∧Q)(LAW OF COMMUTATIVE)

Hence it is proved that Left Hand Side is logically equivalent to Right Hand Side. □

The proposed methodology is proved by Theorem 1, which demonstrate that all authentication conditions of the Blockchain are met if and only if, the user is authenticated. Blockchain authentication provides a robust mechanism by authenticating any user when all said conditions are fulfilled, and even an attacker cannot change any data in any Blockchain node.

## 4. Experimentation Results

Experimental tests were carried out with the formal method tool Scyther. The tool facilitates to conduct experiment with bounded as well as unbounded number of sessions. Scyther automatically verifies all the security protocols. Scyther’s adversary model is based on the Dolev–Yao model [47]. Scyther creates an attack graph on detecting an attack. It is based on the pattern-refinement algorithm that gives the brief and to the point representation of sets traces (infinite) [48]. Scyther allows to specify all the security requirements in terms of claim events [49]. Scyther contains four claim events: Alive, Nisynch, Secret and Commitment [50]. The process of achieving the intended communication with some events is described as “Alive”. Nisynch stands for non-injective synchronization which ensure that the intended sender sends all messages received by the receiver in a synchronized manner. Commitment is a promise that is made by one party to the other. It is confidential user data that is achieved by using Secret.

**Figure 2:**
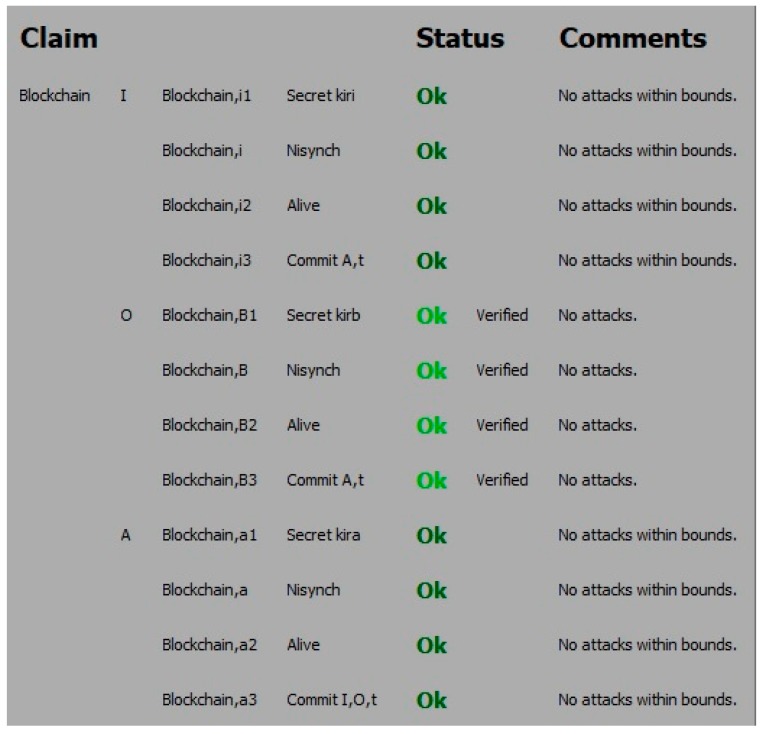
The output for the Scyther claim test for I, B and A.

The results are shown in Figure 2. The status Ok means there were no attacks within bounds. The nonce is a session variable which ensures no old value reused. Scyther is used to verify these security requirements. It can be seen from Figure 2 that all four claims have achieved and verified. The comparisons between the proposed scheme and other related authentication schemes are presented in Table 3.

It can be concluded that the proposed solution resisted the well-known primary attacks and guaranteed the primary security requirements, and highy efficient in operation.

**Figure 3:**
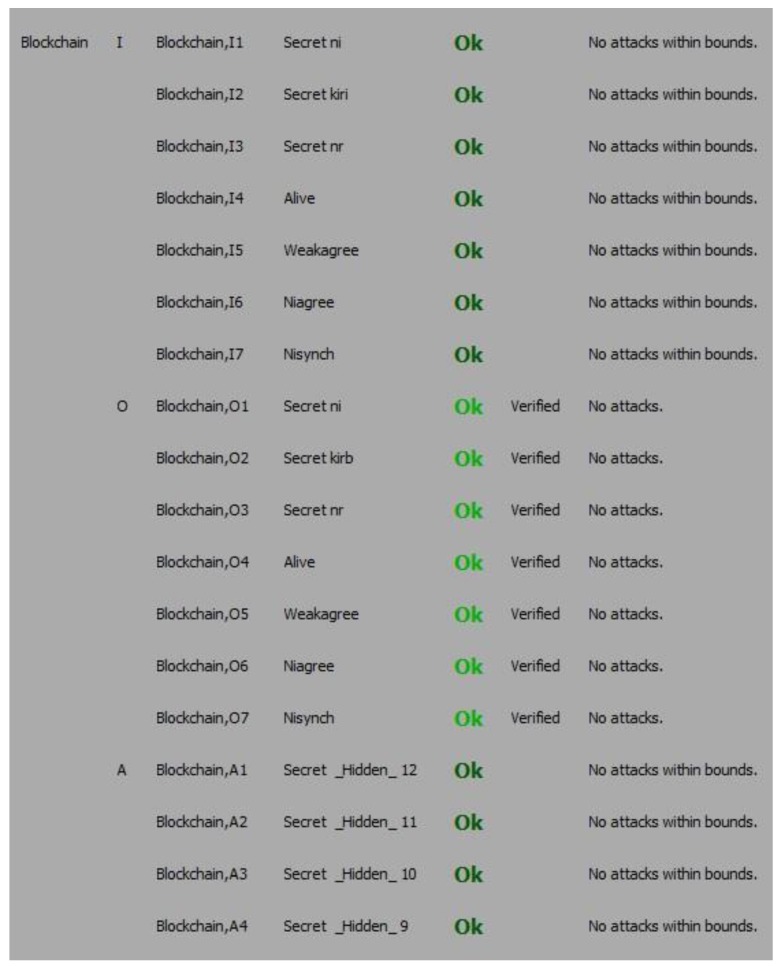
The verification result of the automatic claim.

From Figure 3, it is proved, that the proposed mechanism for user authentication withstands all possible attacks and no attack was found within its bounds. It also verifies the working of protocol has been successfully achieved by the automatic claims.

## 5. Conclusions

The research paper comprehensively explained the security flaw’s existing in the cloud environment and has proved how insiders, as well as outsiders, can bypass the authentication system in cloud databases. Furthermore, a Blockchain authentication mechanism for counterfeiting insider as well as outsider attacks is proposed. Blockchain provides numerous benefits in the case of authentication as it is tamperproof and user data is stored in a secured list. Blockchain is a promising technology finds new areas to be explored in coming time [51,52,53,54].

The proposed system is tested using Scyther formal system tool against various attacks to evaluate the performance. The results prove that the proposed system is highly efficient and successful in mitigating various outsider and insider threat’s. It also enhances the security of the cloud environment by identifying all sorts of possible attacks. Moreover, the working of the protocol is also verified based on the four claims and Scyther proved that proposed protocol is robust enough for real-time working environments. 

User privileges allow granting of a different set of authorization rules for a different set of users.In future work, work will focus more on authorization policies to club with authentication rules so that required user privileges can be granted and user access control can be enhanced by allowing user control and monitoring. 

## Figures and Tables

**Table 1:** Comparison of different techniques against an insider attack.

Features Available	Wu et al. [1]	Moon et al. [8]	Yaseen et al. [9,10,11]	Dou et al. [12]	Shaghaghi et al. [13]	Chattopadhyay et al. [14]	Baracaldo et al. [15]
Insider behavior/Activity Analysis	No	Yes	Yes	Yes	Yes	Yes	Yes
Modification of Authorization rules based on Insider Activity Analysis	No	Yes	Yes	Yes	Yes	No	Yes
User-Machine integrity Dependency	No	No	No	Yes	No	No	No
Authentication of Insider	No	No	No	No	No	No	No
The encryption used on User Data before querying on cloud	Yes	No	No	No	No	No	No

**Table 2:** Comparison of different authentication techniques for an outsider user.

Features Available	Tsai et al. [16]	Yang et al. [17]	Kumari et al. [18]	Shajina and Varalakshmi [19]	Anakath et al. [20]	Chaudhary et al. [21]	Kumar et al. [22]	Neha and Chatterjee [23]
Authentication Type	Three factor	Two Factor	Multi-Factor	Two Factor	Multi-Factor	Three factor	Biometric	Biometric
Single sign-on	Yes	Yes	No	Yes	No	Yes	No	No
Cryptography Algorithm used	Yes	Yes	Yes	Yes	Yes	Yes	Yes	No
Clustering Algorithm Used	No	No	No	No	No	No	No	Yes
Suitable for Resource constraint IOT	No	No	Yes	No	No	No	No	No
Mutual Authentication	Yes	No	Yes	Yes	No	Yes	Yes	Yes
Multi Owners Authentication	No	No	No	Yes	No	No	No	No
Distributed Ledger Based Authentication	No	No	No	No	No	No	No	No

**Table 3:** The security comparison of the proposed scheme and other related authentication scheme’s.

Attacks	Proposed Blockchain Authentication Mechanism	Tsai et al. [16]	Yang et al. [17]	Shajina and Varalakshmi [19]	Anakath et al. [20]	Chaudhary et al. [21]
Resist of-line password Guessing attack	Yes	Yes	Yes	No	Yes	Yes
Prevent replay attack	Yes	Yes	Yes	Yes	Yes	Yes
Minimize DoS attack during the authentication process	Yes	Yes	Yes	Yes	Yes	Yes
Prevent insider attack	Yes	No	No	No	No	No
Prevent impersonation attack	Yes	No	Yes	Yes	Yes	Yes
